# Characteristics of exceptional responders to lenalidomide-based therapy in multiple myeloma

**DOI:** 10.1038/bcj.2015.91

**Published:** 2015-10-23

**Authors:** T Vu, W Gonsalves, S Kumar, A Dispenzieri, M Q Lacy, F Buadi, M A Gertz, S V Rajkumar

**Affiliations:** 1 Mayo Medical School, Mayo Clinic, Rochester, MN, USA; 2 Division of Hematology, Mayo Clinic, Rochester, MN, USA

## Abstract

We studied all patients at our institution with a diagnosis of multiple myeloma (MM), from 1 January 2004 to 1 July 2009, who received lenalidomide–dexamethasone (Rd) as initial therapy and had a time to progression of 72 months or longer. Of 240 patients, we identified 33 exceptional responders. Twenty-five patients received primary therapy with Rd and eight patients received Rd induction followed by early stem cell transplantation (SCT). Seven of the eight patients who received SCT did not receive maintenance therapy; one patient received 9 months of lenalidomide post transplant. Fifteen (45%) patients had known clonal plasma cell disorder before the diagnosis of MM. The dominant mode of clinical presentation was with lytic lesions in 28 patients. Of those with informative cytogenetics (*n*=24), trisomies were present in 19 (79%), including one patient with concurrent trisomies and t(11;14). Overall, 21 of 24 patients (88%) had either trisomies or t(11;14). None of these exceptional responders had high-risk cytogenetic features at baseline. Twenty-five patients (76%) had a complete response (CR), whereas eight patients (24%) achieved the exceptional response state without ever achieving a CR. We identify a cohort of exceptional responders to Rd-based therapy, representing ~10–15% newly diagnosed MM patients with normal renal function.

## Introduction

Multiple myeloma (MM) is a plasma cell malignancy and part of a spectrum of clonal plasma cell disorders.^1^ The prognosis of MM varies dramatically based on host characteristics, stage, disease biology and response to therapy.^2, 3, 4^ Host characteristics that affect outcome include age, performance status, renal function and comorbidities.^5^ Stage provides a rough estimate of tumor burden and is assessed in MM using the Durie–Salmon Staging System^6^ and the International Staging System.^7^Disease biology is characterized by the underlying molecular cytogenetic classification of MM, as well as additional cytogenetic abnormalities that are acquired with clonal evolution.^4^ Thus, molecular subtypes translocation t (4;14), t 14;16 and 14;20 are associated with adverse prognosis; deletion 17p typically acquired during the course of disease progression is also associated with aggressive disease biology.^3^ Besides cytogenetic markers, disease biology can also be reflected by the presence of circulating plasma cells and lactate dehydrogenase levels. Recently, the Revised International Staging System has combined disease stage and biology, which often overlap into one prognostic system.^8^

Host characteristics, stage and disease biology are known at baseline and can help with selection of therapy.^2, 5^ However, the fourth determinant of prognosis, response to therapy, is known only after therapy has already been administered. Several studies have examined the association between depth of response and outcome, as well as characteristics of patients with deep (complete) responses.^9, 10, 11, 12^ Of late, there is growing interest in identifying exceptional responders to therapy in terms of duration of response.^13, 14, 15^ Understanding the characteristics of exceptional responders to MM therapy can help us learn more about disease subtypes, biology and even identify new therapeutic strategies.

Lenalidomide is an important component of modern myeloma therapy, both in the frontline and relapsed refractory setting.^16, 17^ The goal of this study was to identify and characterize exceptional responders to lenalidomide-based initial therapy.

## Patients and methods

We identified all patients with a diagnosis of MM, from 1 January 2004 to 1 July 2009, who received lenalidomide–dexamethasone (Rd) as initial therapy for MM at Mayo Clinic, Rochester, MN, USA. We then identified patients with a time to progression (TTP) of 72 months or longer. This included patients who received Rd as primary therapy as well as patients who took Rd as induction and then proceeded to early (<12 months from diagnosis) autologous stem cell transplantation (SCT). Patients who stopped Rd because of any reason before progression (patient choice, physician choice, toxicity and soon) but resumed lenalidomide or Rd due to progression off therapy were also included in the cohort. Patients who received any other anti-MM drug and patients who underwent tandem transplantation during the 72 months following diagnosis were excluded. Patients with concomitant amyloidosis were excluded. Patients who received focal radiation to symptomatic sites at the time of diagnosis of MM were included only if the radiation was administered at the time of initial diagnosis before initiation of systemic therapy. The study was approved by the Institutional Review Board of the Mayo Clinic.

The electronic medical records, including demographic data, clinical notes, laboratory tests, imaging studies and pathologic reports were reviewed to verify therapy, response to treatment and to identify progression events. Relevant laboratory data including bone marrow plasma cell percentage, cytogenetics based on fluorescent *in situ* hybridization and conventional metaphase karyotyping, serum and urine M protein, free light chain ratio, hemoglobin, calcium and creatinine were abstracted for analysis from the electronic medical records and the MM clinical database at the Mayo Clinic.

Patients were classified into the primary molecular subtypes of MM using the classification proposed by Kumar *et al.*^4^ Molecular classification was assessed by fluorescent *in situ* hybridization studies in all patients, except one patient in whom the classification of trisomies was made by baseline metaphase cytogenetics that showed hyperdiploidy and trisomies. All fluorescent *in situ* hybridization studies were performed for clinical purposes at the Mayo Clinic as previously described.^18, 19^ For molecular classification, categories were assigned regardless of when these abnormalities were detected in the course of the disease, including after therapy.^20^ Deletion 17p was only considered if it was present at initial diagnosis. Patients were considered to have high-risk disease if fluorescent *in situ* hybridization studies demonstrated one of the following abnormalities: t(4;14), t(14;16), t(14;20) or loss of p53 gene locus (del17p or monosomy 17).

The *χ*^2^-test was used to compare nominal values. TTP was measured from the date of diagnosis of MM until disease progression. Kaplan–Meier analysis was performed to generate progression and survival curves.

## Results

Of 240 patients who were treated with Rd for newly diagnosed MM during the inclusion period for the study (155 primary Rd and 85 Rd induction followed by early SCT), we identified 33 exceptional responders (14%). Median follow-up was 85 months, range: 73–137 months. Most (23 patients) started therapy with low-dose dexamethasone; the remaining started with the pulsed high-dose dexamethasone regimen but decreased to low-dose dexamethasone after the initial few cycles. Two patients received radiation to one symptomatic site at the time of initial diagnosis. The International Staging System Stage was I in 15 patients, II in 14 patients, III in 3 patients and unevaluable in 1 patient. Durie–Salmon Stage was I in 1 patient, II in 11 patients and III in 21 patients. Detailed patient characteristics with respect to baseline clinical features and laboratory parameters are provided in Table 1.

Fifteen of 33 patients (45%) had a known clonal plasma cell proliferative disorder before the diagnosis of MM, including monoclonal gammopathy of undetermined significance (five patients), smoldering MM (four patients), solitary plasmacytoma (five patients) and two concurrent plasmacytomas (one patient). The dominant mode of presentation at diagnosis was with lytic lesions (28 patients), anemia (4 patients) and hypercalcemia (1 patient). None had extramedullary plasmacytomas at presentation.

Twenty-seven patients (82%) had a measurable level of serum M protein (⩾1 g/dl). Among the six patients without a measurable serum M protein, five had free light chain-only type of MM. The remaining patient had an oligo-secretory IgA κ-MM. The serum M protein level was ⩾3 g/dl in 16 patients (48%) and ⩾4 g/dl in 9 patients (27%). In contrast, the urine M protein was ⩾2 g/24 h in only one patient; the involved serum free light chain level was <1000 mg/dl in all patients. Bone marrow plasma cell percentage was 60% in 12 patients (36%) and 80% in 2 patients (6%).

Table 2 provides the molecular classification of MM based on cytogenetic studies performed on bone marrow examination in this cohort. Of those with informative cytogenetics (*n*=24), a hyperdiploidy/trisomy was present in 19 of 24 patients (79%) including the 1 patient with concurrent trisomies and t(11;14). The t(11;14) translocation was seen in three patients (8%). Overall, 21 of 24 patients (88%) had either trisomies or t(11;14). Isolated monosomy 13 (in the absence of any immunoglobulin heavy chain translocation or trisomy) was seen in 3 patients (8%). Of note, none of these exceptional responders had any high-risk cytogenetic features at baseline. Abnormalities on metaphase cytogenetics were seen in 4 of 27 patients (15%) on whom this test was done; in three patients the dominant abnormality was hyperdiploidy and the remaining patient had del 13q. When examined by the myeloma defining event at diagnosis, 19 of 20 patients (95%) with bone disease and informative cytogenetics had either trisomies or t(11;14). Of the 4 patients who presented with anemia as the myeloma defining event, 2 had isolated monosomy 13 and the other 2 had trisomies.

The response to therapy in this cohort is shown in Table 3 and represents the best response achieved anytime during therapy. Twenty-four patients (76%) had a complete response (CR); however, eight patients (24%) achieved the exceptional response state without ever achieving a CR, including three patients who only reached a partial response status and continued to have a measurable M protein. Among patients who achieved CR (*n*=25), the median time to CR was 15 months (range: 1–87 months); the time to CR was >12 months in 13 patients (52%) and >36 months in 5 patients (15%).

The exceptional responders could be classified into four groups (Table 4): primary therapy with Rd until progression (*n*=9), primary therapy with Rd but stopped treatment before progression (*n*=11), primary therapy with Rd stopped before progression and resumed later at first progression (*n*=5) and Rd induction followed by early SCT (*n*=8). None of the patients treated with Rd followed by early SCT received lenalidomide maintenance, except one patient who received lenalidomide for 9 months starting day 100 post transplant. No appreciable differences were noticed between the four groups based on the key baseline variables. However, the time to CR differed; median times were 6 months (primary Rd stopped therapy before progression), 14 months (primary therapy with Rd stopped before progression and resumed later at first progression), 15 months (Rd induction followed by early SCT) and 31 months (primary therapy with Rd until progression). Among patients who were in the primary Rd but stopped treatment before progression, the median duration of therapy was 37 months (range: 6–102 months); median TTP after stopping was 63 months.

Three of the 33 patients have died. The cause of death was MM (2 patients) and unrelated infection (1 patient). The estimated 10-year survival rate was 85%. Twenty-three of 33 patients are alive and are progression free (69.7%), including 18 (54.5%) patients who can be considered disease free (sustained CR). Nine patients have progressed and one patient died without disease progression. Follow-up beyond the first progression event (which by definition would occur only after 72 months from initial diagnosis) is too short to analyze response to subsequent therapy. Among patients who progressed, the estimated post-progression 3-year survival rate was 89%.

## Discussion

In this study we have identified and characterized a cohort of exceptional responders to lenalidomide-based therapy. We chose a TTP of 72 months, which is approximately three times the TTP expected with primary Rd.^21^ The presentation and distribution of several clinical features in exceptional responders (Table 1) appear to be similar to those seen in MM in general, including gender, hemoglobin concentration, monoclonal protein type, serum M protein concentration and bone marrow involvement.^22^ The proportion of patients with light-chain MM was also as expected, although overall urine M protein levels was ⩾2 g/dl in one patient and the serum involved free light chain levels were <1000 mg/dl in all patients. However, exceptional responders do appear to have some unique features of interest and represent the main findings of this study. First, a substantial proportion (45%) had a history of antecedent plasma cell disorder (monoclonal gammopathy of undetermined significance (MGUS), smoldering MM (SMM) or plasmacytoma), including plasmacytoma in six patients (18%). In a previous study at the Mayo Clinic, an antecedent plasma cell disorder was found in 34% of patients with MM and only 5% had a prior history of plasmacytoma.^22^ As all MM is preceded by a premalignant phase, the probability that an antecedent plasma cell disorder will be clinically detected will be proportionate to the duration spent in that phase. Thus, more indolent disease evolution with a longer premalignant course is more likely to be detected. Thus, our finding of a greater-than-expected number of patients with a known antecedent plasma cell disorder may indicate that exceptional responders have more favorable disease biology at baseline. In fact, Kyle *et al.*^22^ have shown that patients with a known history of MGUS and SMM before MM diagnosis have a better outcome than patients who present with *de novo* MM.

Second, most exceptional responders to Rd-based therapy had the trisomic type of MM (79%). The remaining had t(11;14) or isolated monosomy 13/del 13q. Trisomies generally constitute ~50% of MM^4^ and, hence, this finding suggests that patients with the trisomic form of MM are the ones in whom an exceptional response to Rd-based (or other immunomodulatory) therapy is likely to be seen. We have reported earlier that the trisomic form of MM is exquisitely sensitive to Rd^23^ and these data are consistent with that observation. None of the exceptional responders had high-risk cytogenetics. Although it is possible that this group is unlikely to have an exceptional response regardless of treatment strategy, it is possible that with other regimens such as bortezomib-based therapy a similar degree of exceptional response in patients with higher-risk cytogenetic categories, especially t(4;14) MM, may be possible.^24^

Third, 85% of patients presented with bone disease as the myeloma defining event, including all but two patients with trisomies. We have previously noted that the more favorable prognosis of trisomic MM may be related to the fact that this subtype has a greater tendency towards bone disease and may thus be labeled as MM earlier than other subtypes, especially t(11;14) or t(4;14).^25^ Thus, early diagnosis and intervention may have had a role in the outcome of these patients and this needs further study.

Fourth, although most patients who stop Rd after a fixed duration of therapy will experience disease progression within a few months,^21^ there is a subset of patients who have an enduring response to Rd even after stopping therapy. In our study, among patients who took primary Rd and stopped early after a median duration of therapy of 3 years, the median TTP after stopping was ~5 years.

Finally, it is important to note that achieving a CR or the rapidity of achieving CR was not essential to be an exceptional responder. Approximately 25% of patients were not in CR, including ~10% of patients who only achieved a partial response with therapy. This indicates that either the residual M protein is arising from a residual non-malignant monoclonal gammopathy of undetermined significance or that it represents a malignant clone that is not capable of proliferation due a microenvironment made inhospitable following therapy.^26^ In either event, this finding should raise some caution to the pursuit of minimal residual disease-negative state, as a proportion of patients may have excellent outcome even without achieving CR. The time to CR also appears to be slower in patients destined to be exceptional responders; median time to CR was 14 months and, more importantly, it was longer than 3 years in 15% of patients. Interestingly, it does appear that the speed at which CR is achieved influences in some way the treatment strategy, with patients who achieve CR sooner going off therapy before disease progression and those who have a slow response tending to stay on therapy until progression.

None of the patients in our cohort of exceptional responders had renal failure at presentation. Such patients may have been systematically excluded, because we do not initiate Rd-based therapy in patients with acute renal failure at diagnosis. However, none of the patients had even a mild elevation of serum creatinine indicates that exceptional responders to Rd-based therapy need to have normal renal function at baseline.

In summary, we identify a cohort of exceptional responders to Rd-based therapy, representing ~10–15% of newly diagnosed MM patients with normal renal function. Approximately 25% of exceptional responders achieved this status without achieving CR. These patients predominantly had the trisomic form of MM and presented with bone disease as their initial myeloma defining event; in such patients, the probability of being an exceptional respond to Rd is likely to be 25–30%. In an era of increasing therapeutic options for MM, these patients will probaby do exceedingly well even after disease progression on Rd. It is also possible that a proportion of these patients are even cured of their MM (residual M protein notwithstanding) with just one line of therapy. If we can accurately identify exceptional responders by biomarkers or genomic approaches, we can spare them the toxicity and costs of additional chemotherapeutic agents. Our next steps are to examine the trisomic form of MM in greater detail, to identify specific factors that will predict exceptional response to Rd-based therapy.

## Figures and Tables

**Table 1:** Baseline characteristics

	*Patients* N*=33*
Age, years, median (range)	59 (32–78)
Age <50 years, *N* (%)	7 (21)
Female sex, *N* (%)	12 (36)
Hemoglobin, median (range), g/dl	11.5 (7.5–15.6)
Hemoglobin <9 gm/dl, *N* (%)	1 (9)
Serum creatinine, median (range), mg/dl	0.9 (0.6–1.3)
Serum calcium, median (range), mg/dl	9.7 (8.5–13)
Calcium >11.0 mg/dl, *N* (%)	2 (7)
Lactate dehydrogenase, median (range), mg/dl	150 (95-206)
Beta-2 microglobulin, median (range), μg/ml	3.73 (1.37–10.9)
Beta-2 microglobulin, >3.5 μg/ml	17 (52)
	
*Monoclonal protein type*	
IgG	22 (67)
IgA	7 (21)
	
FLC only	4 (12)
Serum monoclonal protein spike, median (range), g/dl	2.8 (0.0–6.0)
Serum monoclonal protein spike, >3.0 g/dl	16 (48)
Urine monoclonal protein spike, median (range), g/dl	0.1 (0.0–4.7)
Not present	12 (36)
Detected on immunofixation only	4 (12)
Measurable but <0.5 g/24 h	12 (33)
⩾0.5 g/24 h	5 (15)
BMPC percentage, median (range)	40 (2–90)
BMPC >60%	12 (36)
	
*Serum FLC assay*	
Involved FLC, median (range), mg/dl	10.2 (0.4–929)
Involved/uninvolved ratio	33.2 (0.938–1206.66)
Involved/uninvolved FLC level⩾100 mg/dl	6 (18)
Involved/uninvolved FLC ratio⩾100	9 (27)

Abbreviations: BMPC, bone marrow plasma cell; FLC, free light chain.

**Table 2:** Distribution of primary cytogenetic categories

*Molecular cytogenetic classification*	*All patients in whom cytogenetic studies were done (*n*=28)*	*Patients with informative cytogenetic results (*n*=24)*
	*No. of patients (%)*	*No. of patients (%)*
Trisomies^a^	19 (68)	18 (75)
t(11;14)(q13;q32)	2 (7)	2 (8)
t(4;14)(p16;q32)	0 (0)	0 (0)
*MAF* translocations [t(14;16)(q32;q23) and t(14;20)(q32;q11)]	0 (0)	0 (0)
Other/unknown IgH translocation partner	0 (0)	0 (0)
Both IgH translocation and trisomies^b^	1(4)	1 (4)
Monosomy13/del(13q) in the absence of IgH translocation or trisomies^c^	3 (11)	3 (13)
Normal or insufficient plasma cells	4 (14)	NA

Abreviations: FISH, fluorescent *in situ* hybridization; NA, not applicable.

Trisomies were detected on baseline FISH studies in 18 of 19 patients (includes 1 patient with tetrasomy 11) and by metaphase cytogenetics in 1 patient.

This patient had t(11;14) and trisomies.

Includes one patient with concurrent monosomy 14.

**Table 3:** Response to therapy

*Response category*	*Patients* N*=33*
	N *(%)*
Complete response	25 (76)
Very good partial response	5 (15)
Partial response	3 (9)

**Table 4:** Baseline characteristics and response according by treatment group

	*Primary Rd until progression (*n*=9)*	*Primary Rd stopped before progression (*n*=11)*	*Primary Rd stopped with progression and restarted (*n*=5)*	*Rd induction followed by SCT (*n*=8)*
Age, years, median (range)	65 (36–70)	60 (40–78)	55 (32–75)	62 (36–73)
Prior plasma cell disorder, *N*	6 (3 MGUS/SMM; 3 plasmacytoma)	3 (3 MGUS/SMM)	3 (2 MGUS/SMM; 1 plasmacytoma)	3 (1 MGUS/SMM; 2 plasmacytoma)
MDE at presentation	Bone, 8 Anemia, 1	Bone, 10 Anemia, 1	Bone, 3 Anemia, 1 Hypercalcemia, 1	Bone, 7 Anemia, 1
Molecular classification, *N*	Trisomies, 5 t(11;14) 1t(11;14) plus trisomy, 1Isolated del 13, 1Normal/insufficient, 1	Trisomies, 6t(11;14), 1 Not done, 4	Trisomies, 3 Normal/insufficient, 1 Not done, 1	Trisomies, 4 Isolated del 13, 2 Normal/insufficient, 2
Best response to therapy	CR, 7 VGPR, 2	CR, 9 VGPR, 1 PR, 1	CR, 2 VGPR, 2 PR, 1	CR, 7 PR, 1

Abbreviations: CR, complete response; MDE, myeloma defining event; MGUS, monoclonal gammopathy of undetermined significance; Rd, lenalidomide–dexamethasone; SCT, stem cell transplantation; SMM, smoldering multiple myeloma.
